# Factors Associated with Non-Adherence to Blood Glucose Testing Among Non-Hispanic Black and Hispanic Men with a History of Prediabetes in the United States

**DOI:** 10.2147/PPA.S589075

**Published:** 2026-06-17

**Authors:** Aaheli Bose, Tyler Prochnow, Caroline D Bergeron, Jeong-Hui Park, Bobbie L Johannes, Ledric D Sherman, Megan S Patterson, Ashley L Merianos, Matthew Lee Smith

**Affiliations:** 1 Department of Health Behavior, School of Public Health, Texas A&M University, College Station, TX, USA; 2 Center for Health Equity and Evaluation Research, Texas A&M University, College Station, TX, USA; 3 Center for Community Health and Aging, Texas A&M University, College Station, TX, USA; 4 LIFE Research Institute, University of Ottawa, Ottawa, Ontario, Canada; 5 Department of Health Disparities Research, Division of Cancer Prevention and Population Science, The University of Texas MD Anderson Cancer Center, Houston, TX, USA; 6 Department of Population Health Sciences, Geisinger, Danville, PA, USA; 7 College of Education, Criminal Justice, and Human Services, University of Cincinnati, Cincinnati, OH, USA; 8 Department of Applied Health Science, School of Public Health, Indiana University, Bloomington, IN, USA; 9 Center for Health by Design, School of Public Health, Indiana University, Bloomington, IN, USA

**Keywords:** prediabetes, blood glucose testing, non-Hispanic Black, Hispanic, men

## Abstract

**Introduction:**

Rates of prediabetes are rising in the United States, especially within communities of color. Without adopting self-care behaviors and adhering to routine screenings, prediabetes is likely to progress to type 2 diabetes. This study examines factors associated with non-adherence to recommended blood glucose testing among non-Hispanic Black and Hispanic men ages ≥40 years with a history of prediabetes.

**Methods:**

Using an internet-delivered survey, data were collected from 769 Black (56.7%) and Hispanic (43.3%) men with a history of prediabetes. Chi-square tests and independent sample-tests were to identify differences across study variables by race/ethnicity and blood glucose testing adherence. A logistic regression model with backward stepwise entry was fitted to assess factors associated with blood glucose testing non-adherence, adjusted for sociodemographic factors, co-morbidities, healthcare interactions, social support, and lifestyle behaviors.

**Results:**

Approximately 11% of participants did not receive a blood glucose test in the past 12 months, and 60.9% self-reported progression from prediabetes to diabetes. Men who self-reported progressing from prediabetes to diabetes were less likely to be non-adherent to blood glucose testing recommendations (Odds Ratio [OR]=0.51, P=0.006). Each additional year of age was associated with lower odds of being non-adherent to blood glucose testing (OR=0.97, P=0.018). On average, men who engaged more with healthcare providers during visits had lower odds of being non-adherent to blood glucose testing (OR=0.87, P<0.001). Men who reported reasons for medication non-adherence (OR=1.68, P=0.043) and used tobacco products (OR=2.39, P<0.001) were more likely to report blood glucose testing non-adherence.

**Conclusion:**

Findings suggest that disease progression, greater engagement with healthcare providers, and improved healthy behaviors may be associated with adherence to blood glucose testing among Black and Hispanic men with a history of prediabetes. Culturally tailored interventions and strategies are needed to improve adherence among male these sub-groups.

## Plain Language Summary

Without adopting self-care behaviors and adhering to recommended screenings, prediabetes is likely to progress to type 2 diabetes. This study examined factors associated with non-adherence to recommended blood glucose testing among non-Hispanic Black and Hispanic men ages ≥40 years with a history of prediabetes. Findings highlight the importance of quality of patient-provider communication and improved healthy behaviors to increase adherence to blood glucose testing among Black and Hispanic men with prediabetes.

## Introduction

Diabetes is a prevalent and chronic medical condition that impacts millions of Americans each year.^1^ Its burden extends beyond health, affecting finances and contributing to numerous health complications.^2^ Recent estimates from the Centers for Disease Control and Prevention^3^ indicate that approximately 14.7% of U.S. adults (38.1 million) have diabetes and about 38% (97.6 million) have prediabetes, a precursor state often preceding the disease.^4^ Disparities exist across populations,^5^,^6^ with non-Hispanic Black and Hispanic individuals experiencing disproportionately higher rates of prediabetes compared to non-Hispanic White individuals,^7^ and with more men (41.0%) than women (32.0%) affected.^3^ Risk factors for prediabetes include a body mass index (BMI) greater than 25, history of gestational diabetes, age over 35,^4^ physical inactivity, hypertension, elevated cholesterol, and other predisposing conditions.

Although racial and ethnic disparities in prediabetes prevalence, diabetes risk factors, and abnormal blood glucose screening have been documented, relatively few studies have focused specifically on adherence to recommended blood glucose testing among middle-aged and older non-Hispanic Black and Hispanic men with a history of prediabetes.^7–9^ This gap is important because men in racial and ethnic minority groups are less likely than non-Hispanic White men to participate in diabetes prevention and management programs, despite experiencing a higher burden of type 2 diabetes risk.^10^ Recent evidence suggests that Hispanic men face unique barriers to diabetes prevention engagement, including limited awareness of prediabetes, skepticism about personal diabetes risk, financial constraints, and concerns about program relevance.^11^

Prediabetes and diabetes are primarily diagnosed with four tests, including hemoglobin A1c (HbA1c), random blood sugar, fasting blood sugar, and oral glucose tolerance test.^12^ Since blood glucose testing is one of the primary methods used to identify abnormal glycemia and monitor risk status over time, adherence to recommended testing is an important component of prediabetes management.^13^,^14^ For adults with prediabetes, routine follow-up testing provides opportunities for early counseling, risk communication, and referral to evidence-based diabetes prevention resources before progression to type 2 diabetes; however, prior studies suggest that prediabetes-related clinical care activities, including diagnosis, counseling, and referral to preventive interventions, remain underused in primary care settings.^15^,^16^

Early detection and intervention in prediabetes are essential to prevent progression to type 2 diabetes. Annual blood glucose testing is strongly recommended for those with prediabetes, while individuals with normal glycemia are screened very three years.^4^,^13^ Evidence-based lifestyle recommendations include smoking cessation, moderated alcohol intake, a balanced diet, regular exercise, adherence to prescribed medications, and achieving a 5–7% reduction in body weight.^17^ Despite the proven effectiveness of these measures, sustained behavioral changes can be difficult.^18^

Barriers to effective self-care and adherence to prediabetes management including routine screenings, may include socioeconomic challenges,^19^,^20^ limited access to healthcare,^3^ communication gaps with healthcare providers,^21^ medication costs,^21^ and inadequate social support.^22^ Regular follow-up and effective patient-provider communication, supported by telehealth and patient portals, are critical to sustained engagement.^23^ Missed appointments reduce opportunities for routine screening and ongoing education.^24^,^25^ These barriers may be reflected in multiple, interrelated adherence behaviors because chronic disease self-management requires patients to integrate clinical recommendations into daily routines while navigating health beliefs, social support, and access to resources.^26^,^27^ For example, medication non-adherence may signal challenges related to habitual behavior, treatment beliefs, self-efficacy, or financial resources.^27^ Tobacco use may represent a biological risk factor for diabetes-related complications and a marker of broader disengagement from preventive healthcare among men with chronic conditions.^15^,^28^ Conversely, active engagement during physician visits may reflect greater personal agency, preparedness, and capacity to navigate clinical recommendations, which are relevant to routine blood glucose testing and chronic disease prevention.^26^,^29^,^30^

The present study focused on non-Hispanic Black and Hispanic men aged 40 years and older because this group falls within clinically emphasized screening age ranges and reflects the parent study’s focus on chronic disease management among middle-aged and older men with chronic conditions. Current recommendations advise testing adults beginning at age 35 years, particularly among those with overweight or obesity, while prior United States Preventive Services Task Force (USPSTF) guidance recommended screening adults aged 40 to 70 years with overweight or obesity as part of cardiovascular risk assessment.^14^,^31^ This focus is also important because Black and Hispanic/Latino adults experience disproportionately high diabetes burden, and men in racial and ethnic minority groups remain underrepresented in diabetes prevention and management programs.^3^,^10^,^11^

Because non-Hispanic Black and Hispanic men face a dual burden of high prediabetes rates and poor diabetes-related outcomes,^5^,^8^,^13^ there is an urgent need to identify modifiable factors that influence adherence to recommended blood glucose testing. This study examines the behavioral, clinical, and healthcare engagement factors associated with non-adherence among non-Hispanic Black and Hispanic men aged 40 and older with a history of prediabetes. Guided by this gap, the present study examined behavioral, clinical, and healthcare engagement correlates of blood glucose testing non-adherence among non-Hispanic Black and Hispanic men with a history of prediabetes. Specifically, we assessed whether self-reported diabetes progression, patient-provider communication, engagement during physician visits, medication adherence patterns, tobacco use, and other health-related factors were associated with non-adherence to annual blood glucose testing. Understanding these factors can inform evidence-based strategies and educational interventions on regular blood glucose monitoring.

## Materials and Methods

### Participants and Procedures

This study employed an internet-delivered, cross-sectional survey design using a non-probabilistic national sample collected through a Qualtrics Online Panel.^32^ The survey was part of a larger parent study examining health indicators, healthcare utilization, and barriers to disease management among non-Hispanic Black and Hispanic men ages 40 years and older who self-reported one or more chronic conditions.^26^ A robust online questionnaire was developed using previously validated items and scales from sources such as the Behavioral Risk Factor Surveillance System and National Study of the Chronic Disease Self-Management Program.^33–35^ The instrument included 105 items, which took approximately 30 minutes to complete. The survey was conducted between September and October 2019. Participants accessed the survey through an electronic link and were required to review and acknowledge an informed consent prior to participation. Participants completed the questionnaire independently online. Data quality assessments were performed by Qualtrics in terms of data completeness, reasonable time to complete, and participant uniqueness.^32^ Further details about the survey instrument and methodology have been published in detail elsewhere.^26^,^29^,^30^,^36–40^ This study complies with the Declaration of Helsinki and was approved by the Texas A&M University Institutional Review Board (ID: 2018–1684).

Of 2029 men who met the inclusion criteria (non-Hispanic Black and Hispanic men aged 40 years or older with one or more chronic conditions) and completed the survey, 47 cases were omitted because they self-identified as both Black and Hispanic. Among these, 958 participants self-reported a prediabetes diagnosis. To omit biases related to healthcare access and interactions with healthcare providers, 189 men were omitted for not reporting a routine check-up with a physician in the past year. The final analytic sample was 769 non-Hispanic Black and Hispanic aged 40 and over with prediabetes who had a routine check-up in the past year.

### Measures

The dependent variable for this study was whether the participants received a blood glucose test in the past 12 months. Participants were asked, “When was the last time you had a blood sugar test?” Response choices were “within the past year,” “within the past two years (more than one year ago),” “within the past three years (more than two years ago),” “within the past four years (more than three years ago),” “within the past five years (more than 4 years ago),” and “five or more years ago.” This dichotomization was selected based on both clinical guidance and the observed response distribution in the analytic sample. The Centers for Disease Control and Prevention (CDC) indicates that individuals with prediabetes will likely be advised to repeat A1C testing every 1 to 2 years;^41^ however, the frequency distribution in the current sample indicated that most participants reported receiving a blood glucose test within the past year. Therefore, we operationalized recent blood glucose testing adherence as having received a blood glucose test within the past 12 months (0=adherent; 1=non-adherent) to distinguish men who reported recent testing from those whose most recent test occurred more than 12 months prior.

#### Self-Reported Progression to Diabetes

Participants were also asked, “Have you ever been told by a doctor or other health professional that you have prediabetes or borderline diabetes?” Response choices were “no” and “yes.” Participants were also asked to self-report all chronic conditions that a healthcare provider told them they had.^26^ The participants were provided with a list of 19 chronic conditions, of which diabetes was an option. Response choices were “no” and “yes.” Given that all participants in the current study self-reported a history of prediabetes, self-reported diabetes was included in the analyses to indicate the progression from prediabetes to diabetes. The progression to diabetes variable in this cross-sectional study was based on self-reported diagnosis from a healthcare professional and not confirmed by clinical testing.

#### Health Indicators

Body mass index (BMI) was calculated as self-reported weight (pounds) divided by height (inches) and multiplied by 703. The resulting BMI scores were categorized for analyses as normal weight (18.5 to 24.99 kg/m^2^), overweight (25 to 29.99 kg/m^2^), and obese (≥ 30 kg/m^2^).^42^ As mentioned above, participants were asked to self-report whether they had been diagnosed with a series of chronic conditions by a healthcare provider. Health conditions relevant to prediabetes and diabetes were included in the analyses (ie., heart disease, high cholesterol, hypertension, and thyroid condition). Each of these variables was binarily coded (no/yes) and analyzed separately.

#### Frequency of Help and Support Needed to Improve Health and Manage Health Problems

The frequency of participants receiving the health and support needed to improve their health and manage health problems was measured using a 5-point scale ranging from “never” (scored 1) to “always” (scored 5).^43^,^44^

#### Physician Quality Conversation and Joint Decision-Making Scale

This 6-item scale asked participants about the extent to which they believed their conversations with physicians were of high quality and included joint decision making.^43^,^44^ Using a 5-point scale with response options ranging from “never” to “always,” participants were asked to report how often their healthcare provider: 1) asked for their ideas about how they can take care of their health problems; 2) made plans to contact them after a visit to see how they are doing; 3) helped them get the appointments they need; 4) asked if they understood how and when to take their prescribed medications, possible medication side effects, and drug interactions; 5) talked to other doctors and nurses who are taking care of them; and 6) asked whether they had help at home to manage their health problems. Items were summed, with possible scores ranging from 6–30. Higher scores indicate higher quality conversation. The Cronbach’s alpha scale value was acceptable for the current sample (α=0.841).

#### Engagement During Physician Visit Scale

This 4-item scale asked participants about their engagement with physicians during healthcare visits.^34^,^35^ Using a 5-point scale with response options of “never” to “always,” participants reported how often: 1) they prepared a list of questions for their doctor; 2) their doctor asked questions about things they want to know and things they do not understand about their treatment; 3) they discussed any personal problems that may be related to their illness; and 4) their doctor asked questions until they clearly understand the purpose for taking each of their medications. The possible scores ranged from 4–20, with higher scores indicating higher engagement during physician visits. The Cronbach’s alpha scale value was acceptable for the current sample (α=0.804).

#### Adherence to Medications

Participants were asked to report on their medication taking. Three items were asked: 1) Do you ever have problems remembering to take your medicine? 2) When you feel better, do you sometimes stop taking your medicine? and 3) Sometimes if you feel worse when you take your medicine, do you stop taking it? Response choices for each item were “no” and “yes.” These items were summed to create a count variable, then dichotomized to identify whether participants had one or more reasons why they did not adhere to their prescribed medications (ie., 0=adherent; 1=one or more reasons for not being adherent).

#### Health-Related Behaviors

Participants were asked to report their use of any tobacco product in the past 30 days and the number of alcoholic beverages consumed weekly.^26^,^33^ Tobacco use was dichotomized to reflect whether the participant had reported using tobacco in the past month (no/yes). Alcohol consumption was dichotomized to reflect if the participant consumed one or more alcoholic beverages in a typical week (no/yes).

#### Sociodemographics

The sociodemographic measures included in this study included race and ethnicity (ie., non-Hispanic Black, Hispanic), age (range 40 to 88 years), educational attainment (ie., high school education or less, some college/2-year degree, 4-year college degree or more), marital status (ie., married/partnered, never married, divorced/separated, widowed), number of persons living in the household (including self), and annual household income level (in mostly $10,000 USD increments).

### Statistical Analyses

Data were analyzed using SPSS 29.0 software (SPSS Inc., Chicago, IL, USA). Descriptive statistics were computed to identify sample characteristics, which were compared by race and ethnicity and blood glucose testing adherence in the past 12 months. Proportional differences were compared using Chi-square tests for categorical variables. Mean differences were compared using independent-sample t-tests for continuous variables. Three binary logistic regression models with backward stepwise entry were fitted to identify factors associated with non-adherence to blood glucose testing (ie., getting a blood glucose test in the past 12 months served as the reference category). Logistic regression with backward stepwise entry was used to identify parsimonious models of significant variables while avoiding potential issues of multicollinearity. One model examined the factors associated with non-adherence for all men who met the inclusion criteria. Then, separate models were fitted to examine the associations among non-Hispanic Black men and Hispanic men independently. For each model, the full and reduced models are presented; however, the reported results focus only on the reduced models. Odds ratios (OR) with corresponding p-values and 95% confidence intervals (CIs) are reported. A p-value <0.05 was used to identify statistically significant associations in all analyses.

## Results

Among the 769 men with self-reported prediabetes, 56.7% were non-Hispanic Black, 43.3% were Hispanic, and 10.8% were non-adherent because they had not received a blood glucose test in the past year. About 61% of participants reported that they progressed from prediabetes to diabetes. On average, participants were aged 57.80 (±9.52) years and resided with 2.54 (±1.45) people in their household. Over half were married/partnered (56.2%), had some college education or a 2-year degree (41.7%) or a 4-year college education (38.4%), and were obese (50.2%). Over 16% of the participants self-reported a heart disease diagnosis, 55.9% reported high cholesterol, 65.7% reported hypertension, and 10.1% reported a thyroid problem. Nearly half (48.0%) of the participants reported one or more reasons for not adhering to their prescribed medications, 30.9% reported using tobacco in the past 30 days, and 59.9% reported drinking one or more alcoholic beverages each week. Table 1 reports the characteristics of the participants in the current sample, which were compared by race and ethnicity and blood glucose adherence in the past year.

**Table 1:** Sample Characteristics by Race and Ethnicity and Blood Glucose Testing

	Race and Ethnicity					Blood Glucose Test in 12 Months			
	Total (n=769)	Black (n=436)	Hispanic (n=333)	χ^2^ or t	P	Got Test (n=686)	No Test (n=83)	χ^2^ or t	P
Progression to Diabetes: No	39.1%	40.1%	37.8%	0.42	0.517	37.5%	53.0%	7.51	0.006
Progression to Diabetes: Yes	60.9%	59.9%	62.2%			62.5%	47.0%		
Non-Hispanic Black	56.7%	--	--	--	--	56.3%	60.2%	0.48	0.490
Hispanic	43.3%	--	--			43.7%	39.8%		
Age	57.80 (±9.52)	58.07 (±9.02)	57.43 (±10.13)	0.91	0.363	58.27 (±9.39)	53.92 (±9.71)	3.97	<0.001
Education Level: High School or Less	19.9%	22.7%	16.2%	12.11	0.002	19.2%	25.3%	4.73	0.094
Education Level: Some College or 2-Year Degree	41.7%	44.0%	38.7%			41.1%	47.0%		
Education Level: 4-Year Degree or More	38.4%	33.3%	45.0%			39.7%	27.7%		
Marital Status: Married/Partnered	56.2%	48.2%	66.7%	33.14	<0.001	56.6%	53.0%	0.60	0.897
Marital Status: Never Married	21.7%	27.3%	14.4%			21.7%	21.7%		
Marital Status: Separated/Divorced	16.9%	17.4%	16.2%			16.6%	19.3%		
Marital Status: Widowed	5.2%	7.1%	2.7%			5.1%	6.0%		
Number of Persons Residing In Household (including Self)	2.54 (±1.45)	2.46 (±1.59)	2.63 (±1.25)	−1.61	0.109	2.53 (±1.46)	2.58 (±1.44)	−0.29	0.771
Annual Household Income (~$10,000 increments)	6.21 (±3.42)	5.87 (±3.35)	6.65 (±3.45)	−3.17	0.002	6.30 (±3.47)	5.43 (±2.82)	2.59	0.011
BMI Category: Normal Weight	15.7%	16.5%	14.7%	0.92	0.633	14.1%	28.9%	12.73	0.002
BMI Category: Overweight	34.1%	32.8%	35.7%			35.1%	25.3%		
BMI Category: Obese	50.2%	50.7%	49.5%			50.7%	45.8%		
Has Heart Disease: No	83.6%	85.3%	81.4%	2.14	0.144	83.2%	86.7%	0.67	0.414
Has Heart Disease: Yes	16.4%	14.7%	18.6%			16.8%	13.3%		
Has High Cholesterol: No	44.1%	45.0%	42.9%	0.31	0.578	44.0%	44.6%	0.01	0.923
Has High Cholesterol: Yes	55.9%	55.0%	57.1%			56.0%	55.4%		
Has Hypertension (High Blood Pressure): No	34.3%	28.9%	41.4%	13.17	<0.001	32.8%	47.0%	6.61	0.010
Has Hypertension (High Blood Pressure): Yes	65.7%	71.1%	58.6%			67.2%	53.0%		
Has Thyroid Problem (e.g., Hyperthyroidism, Hypothyroidism): No	89.9%	52.5%	37.3%	8.68	0.003	80.2%	9.6%	0.05	0.823
Has Thyroid Problem (e.g., Hyperthyroidism, Hypothyroidism): Yes	10.1%	7.3%	13.8%			10.1%	10.8%		
Get Help or Support to Improve Health/Manage Health Problems	3.79 (±1.03)	3.85 (±0.99)	3.71 (±1.07)	1.87	0.062	3.82 (±1.02)	3.53 (±1.03)	2.41	0.016
Physician Quality Conversation and Joint Decision Making Scale	19.49 (±5.38)	19.87 (±5.26)	19.00 (±5.50)	2.23	0.026	19.65 (±5.35)	18.23 (±5.47)	2.27	0.023
Engagement During Physician Visit Scale	14.71 (±3.28)	14.78 (±3.26)	14.62 (±3.32)	0.68	0.498	14.85 (±3.21)	13.55 (±3.65)	3.41	<0.001
Reasons Not Adhere to Medications: 0 Reasons	52.0%	52.1%	52.0%	0.00	0.975	54.1%	34.9%	10.87	<0.001
Reasons Not Adhere to Medications: 1+ Reasons	48.0%	47.9%	48.0%			45.9%	65.1%		
Use Any Tobacco Product in Past 30 Days: No	69.1%	64.2%	75.4%	10.99	<0.001	71.9%	45.8%	23.57	<0.001
Use Any Tobacco Product in Past 30 Days: Yes	30.9%	35.8%	24.6%			28.1%	54.2%		
Drink 1+ Alcoholic Beverage Per Week: No	40.1%	39.0%	41.4%	0.47	0.492	41.0%	32.5%	2.19	0.139
Drink 1+ Alcoholic Beverage Per Week: Yes	59.9%	61.0%	58.6%			59.0%	67.5%		

When comparing the sample by race and ethnicity, a larger proportion of Hispanic men had higher educational levels (χ^2^=12.11, P=0.002) and were married/partnered (χ^2^=33.14, P<0.001). On average, Hispanic men reported higher annual household incomes (t=−3.17, P=0.002). A larger proportion of non-Hispanic Black men self-reported hypertension (χ^2^=13.17, P<0.001), while a larger proportion of Hispanic men reported a thyroid problem (χ^2^=8.68, P=0.003). Relative to Hispanic men, a larger proportion of non-Hispanic Black men used a tobacco product in the past 30 days (χ^2^=10.99, P<0.001).

When comparing the sample by blood glucose testing non-adherence in the past 12 months, a larger proportion of men who progressed from prediabetes to diabetes were less likely to be non-adherent to blood glucose testing (χ^2^=7.51, P<0.006). On average, non-adherent men were younger (t=3.97, P<0.001) and had lower annual household income levels (t=2.59, P=0.011). A larger proportion of men of normal weight were non-adherent to blood glucose testing (χ^2^=12.73, P=0.002). A smaller proportion of men with hypertension were less likely to be non-adherent to blood glucose testing (χ^2^=6.61, P=0.010). On average, men who were non-adherent to blood glucose testing reported less help/support to improve their health and manage their health problems (t=2.41, P=0.016), lower quality conversations and joint decision making with physicians (t=2.27, P=0.023), and lower engagement during physician visits (t=3.41, P<0.001). A larger proportion of men who reported one or more reasons they do not adhere with prescribed medications (χ^2^=10.87, P<0.001) and used tobacco in the past 30 days (χ^2^=23.57, P<0.001) were non-adherent to blood glucose testing.

Table 2 reports the logistic regression models that examined non-adherence to blood glucose testing among non-Hispanic Black and Hispanic men. As seen in the reduced model, compared to men with prediabetes, men who progressed from prediabetes to diabetes were significantly less likely to not adhere to blood glucose testing (OR=0.51, P=0.006). Each additional year of age lowered the odds of non-adherence to blood glucose testing (OR=0.97, P=0.018). Higher engagement during physician visits was associated with lower odds of being non-adherent to blood glucose testing (OR=0.87, P<0.001). Men who reported having one or more reasons for medication non-adherence (OR=1.68, P=0.043) and used tobacco in the past 30 days (OR=2.39, P<0.001) were significantly more likely to be non-adherent to blood glucose testing, respectively.

**Table 2:** Factors Associated with Blood Glucose Testing Non-Adherence - All Men (n=769)

	Full Model						Reduced Model (13 Iterations)					
	B	S.E.	P	OR	95% CI		B	S.E.	P	OR	95% CI	
					Lower	Upper					Lower	Upper
Progression to Diabetes (vs. Not)	−0.72	0.25	0.005*	0.49	0.30	0.80	−0.67	0.25	0.006*	0.51	0.32	0.83
Hispanic (vs. Non-Hispanic Black)	−0.25	0.27	0.348	0.78	0.46	1.32						
Age	−0.04	0.02	0.012*	0.96	0.93	0.99	−0.03	0.01	0.018*	0.97	0.94	0.99
Education Level: High School or Less	--	--	--	1.00	--	--						
Education Level: Some College or 2-Year Degree	0.13	0.33	0.695	1.14	0.60	2.15						
Education Level: 4-Year Degree or More	−0.16	0.38	0.682	0.85	0.40	1.82						
Marital Status: Married/Partnered	--	--	--	1.00	--	--						
Marital Status: Never Married	−0.49	0.37	0.183	0.61	0.30	1.26						
Marital Status: Separated/Divorced	−0.26	0.37	0.473	0.77	0.37	1.58						
Marital Status: Widowed	−0.25	0.59	0.672	0.78	0.25	2.47						
Number of Persons Residing In Household (including Self)	−0.08	0.10	0.434	0.92	0.75	1.13						
Annual Household Income (~$10,000 increments)	−0.06	0.05	0.193	0.94	0.85	1.03						
BMI Category: Normal Weight	--	--	--	1.00	--	--						
BMI Category: Overweight	−0.67	0.36	0.064	0.51	0.25	1.04						
BMI Category: Obese	−0.40	0.33	0.219	0.67	0.35	1.27						
Has Heart Disease (vs. No)	−0.27	0.37	0.472	0.77	0.37	1.59						
Has High Cholesterol (vs. No)	0.32	0.26	0.226	1.38	0.82	2.31						
Has Hypertension (High Blood Pressure) (vs. No)	−0.54	0.27	0.047*	0.58	0.34	0.99	−0.45	0.25	0.074	0.64	0.39	1.05
Has Thyroid Problem (e.g., Hyperthyroidism, Hypothyroidism) (vs. No)	0.40	0.42	0.335	1.50	0.66	3.41						
Get Help or Support to Improve Health/Manage Health Problems	0.00	0.14	0.973	1.00	0.77	1.31						
Physician Quality Conversation and Joint Decision Making Scale	−0.03	0.03	0.307	0.97	0.92	1.03						
Engagement During Physician Visit Scale	−0.11	0.04	0.008*	0.89	0.82	0.97	−0.14	0.04	<0.001*	0.87	0.81	0.94
1+ Reasons Not Adhere to Medications (vs. 0 Reasons)	0.44	0.27	0.104	1.54	0.91	2.61	0.52	0.26	0.043*	1.68	1.02	2.79
Use Any Tobacco Product in Past 30 Days (vs. No)	0.71	0.28	0.012*	2.03	1.17	3.54	0.87	0.25	<0.001*	2.39	1.47	3.91
Drink 1+ Alcoholic Beverage Per Week (vs. No)	0.12	0.28	0.680	1.12	0.65	1.95						
Nagelkerke R Square = 0.178							Nagelkerke R Square = 0.148					

**Notes**: Reference Group: Having Blood Glucose Test in Past Year. * Indicates statistically significant associations.

Table 3 reports the logistic regression models that examined non-adherence to blood glucose testing among non-Hispanic Black men. As seen in the reduced model, compared to men of normal weight, overweight men were less likely to be non-adherent to blood glucose testing (OR=0.35, P=0.026). Each additional unit on the Engagement During Physician Visit Scale was associated with lower odds of being non-adherent to blood glucose testing (OR=0.89, P=0.019). Men who reported having one or more reasons for medication non-adherence (OR=2.28, P=0.017) and used tobacco in the past 30 days (OR=3.06, P=0.001) were significantly more likely to be non-adherent to blood glucose testing, respectively.

**Table 3:** Factors Associated with Blood Glucose Testing Non-Adherence - Non-Hispanic Black Men (n=436)

	Full Model						Reduced Model (12 Iterations)					
	B	S.E.	P	OR	95% CI		B	S.E.	P	OR	95% CI	
					Lower	Upper					Lower	Upper
Progression to Diabetes (vs. Not)	−0.69	0.34	0.044*	0.50	0.26	0.98	−0.58	0.33	0.073	0.56	0.29	1.06
Age	−0.03	0.02	0.132	0.97	0.92	1.01						
Education Level: High School or Less	--	--	--	1.00	--	--						
Education Level: Some College or 2-Year Degree	0.17	0.41	0.684	1.18	0.53	2.66						
Education Level: 4-Year Degree or More	−0.21	0.52	0.683	0.81	0.30	2.23						
Marital Status: Married/Partnered	--	--	--	1.00	--	--						
Marital Status: Never Married	−0.77	0.48	0.107	0.46	0.18	1.18						
Marital Status: Separated/Divorced	−0.86	0.55	0.116	0.42	0.15	1.24						
Marital Status: Widowed	−1.18	0.81	0.148	0.31	0.06	1.52						
Number of Persons Residing In Household (including Self)	−0.17	0.14	0.245	0.84	0.64	1.12						
Annual Household Income (~$10,000 increments)	−0.08	0.07	0.253	0.92	0.80	1.06						
BMI Category: Normal Weight	--	--	--	1.00	--	--	--	--	--	1.00	--	--
BMI Category: Overweight	−0.97	0.51	0.056	0.38	0.14	1.03	−1.05	0.47	0.026*	0.35	0.14	0.88
BMI Category: Obese	−0.17	0.43	0.697	0.85	0.37	1.96	−0.29	0.40	0.463	0.75	0.35	1.62
Has Heart Disease (vs. No)	−0.09	0.53	0.863	0.91	0.32	2.59						
Has High Cholesterol (vs. No)	0.37	0.36	0.297	1.45	0.72	2.93						
Has Hypertension (High Blood Pressure) (vs. No)	−0.66	0.38	0.086	0.52	0.25	1.10	−0.66	0.34	0.050	0.51	0.26	1.00
Has Thyroid Problem (e.g., Hyperthyroidism, Hypothyroidism) (vs. No)	0.33	0.66	0.617	1.39	0.38	5.01						
Get Help or Support to Improve Health/Manage Health Problems	0.10	0.19	0.622	1.10	0.75	1.61						
Physician Quality Conversation and Joint Decision Making Scale	−0.04	0.04	0.351	0.97	0.90	1.04						
Engagement During Physician Visit Scale	−0.10	0.06	0.077	0.90	0.80	1.01	−0.12	0.05	0.019*	0.89	0.80	0.98
1+ Reasons Not Adhere to Medications (vs. 0 Reasons)	0.84	0.37	0.022*	2.31	1.13	4.74	0.82	0.35	0.017*	2.28	1.16	4.48
Use Any Tobacco Product in Past 30 Days (vs. No)	1.01	0.39	0.010*	2.76	1.28	5.95	1.12	0.34	0.001*	3.06	1.58	5.94
Drink 1+ Alcoholic Beverage Per Week (vs. No)	0.33	0.40	0.405	1.40	0.64	3.07						
Nagelkerke R Square = 0.227							Nagelkerke R Square = 0.185					

**Notes**: Reference Group: Having Blood Glucose Test in Past Year. * Indicates statistically significant associations.

Table 4 reports the logistic regression models that examined non-adherence to blood glucose testing among Hispanic men. As seen in the reduced model, men who progressed from prediabetes to diabetes were significantly less likely to be non-adherent to blood glucose testing compared to men with prediabetes (OR=0.43, P=0.027). Each additional year of age was associated with lower odds of being non-adherent to blood glucose testing (OR=0.95, P=0.016). Each additional unit on the Engagement During Physician Visit Scale reduced the odds of being non-adherent to blood glucose testing (OR=0.87, P=0.010).

**Table 4:** Factors Associated with Blood Glucose Testing Non-Adherence - Hispanic Men (n=333)

	Full Model						Reduced Model (15 Iterations)					
	B	S.E.	P	OR	95% CI		B	S.E.	P	OR	95% CI	
					Lower	Upper					Lower	Upper
Progression to Diabetes (vs. Not)	−0.76	0.41	0.067	0.47	0.21	1.06	−0.85	0.38	0.027*	0.43	0.20	0.91
Age	−0.05	0.03	0.047*	0.95	0.90	1.00	−0.05	0.02	0.016*	0.95	0.92	0.99
Education Level: High School or Less	--	--	--	1.00	--	--						
Education Level: Some College or 2-Year Degree	0.18	0.58	0.752	1.20	0.39	3.75						
Education Level: 4-Year Degree or More	−0.08	0.64	0.899	0.92	0.26	3.24						
Marital Status: Married/Partnered	--	--	--	1.00	--	--						
Marital Status: Never Married	−0.49	0.69	0.477	0.61	0.16	2.36						
Marital Status: Separated/Divorced	0.25	0.53	0.638	1.28	0.46	3.60						
Marital Status: Widowed	1.05	1.07	0.324	2.87	0.35	23.16						
Number of Persons Residing In Household (including Self)	−0.01	0.17	0.958	0.99	0.71	1.38						
Annual Household Income (~$10,000 increments)	−0.08	0.07	0.287	0.92	0.80	1.07						
BMI Category: Normal Weight	--	--	--	1.00	--	--						
BMI Category: Overweight	−0.20	0.62	0.753	0.82	0.24	2.79						
BMI Category: Obese	−0.74	0.61	0.227	0.48	0.14	1.59						
Has Heart Disease (vs. No)	−0.15	0.58	0.796	0.86	0.28	2.68						
Has High Cholesterol (vs. No)	0.35	0.45	0.439	1.42	0.59	3.43						
Has Hypertension (High Blood Pressure) (vs. No)	−0.51	0.42	0.229	0.60	0.26	1.38						
Has Thyroid Problem (e.g., Hyperthyroidism, Hypothyroidism) (vs. No)	0.37	0.60	0.539	1.45	0.44	4.71						
Get Help or Support to Improve Health/Manage Health Problems	−0.11	0.20	0.596	0.90	0.60	1.34						
Physician Quality Conversation and Joint Decision Making Scale	−0.01	0.04	0.755	0.99	0.90	1.08						
Engagement During Physician Visit Scale	−0.14	0.07	0.041*	0.87	0.76	0.99	−0.14	0.06	0.010*	0.87	0.78	0.97
1+ Reasons Not Adhere to Medications (vs. 0 Reasons)	−0.15	0.44	0.724	0.86	0.36	2.02						
Use Any Tobacco Product in Past 30 Days (vs. No)	0.41	0.45	0.372	1.50	0.62	3.65						
Drink 1+ Alcoholic Beverage Per Week (vs. No)	−0.37	0.44	0.407	0.69	0.29	1.65						
Nagelkerke R Square = 0.181							Nagelkerke R Square = 0.111					

**Notes**: Reference Group: Having Blood Glucose Test in Past Year. * Indicates statistically significant associations.

## Discussion

This study identified factors associated with non-adherence to annual blood glucose testing in a large sample of non-Hispanic Black and Hispanic men with prediabetes, a group known to be at high risk but often underrepresented in adherence research.^9^ In a diverse sample of 769 men with prediabetes, approximately 11% of men did not meet the recommended testing frequency, highlighting an important gap in preventive care.^9^,^13^,^15^ The findings suggest four primary factors were significantly associated with testing non-adherence: progression to type 2 diabetes, quality of patient-provider communication with shared decision-making, medication adherence patterns, and tobacco use.

Men who had progressed from prediabetes to diabetes demonstrated greater adherence to blood glucose testing, consistent with clinical guidelines recommending more frequent monitoring once diabetes is diagnosed.^42^ This higher adherence may reflect personalized diabetes management and increased patient awareness following disease progression.^45^,^46^ These adherence differences between groups emphasize the need for early and sustained engagement with individuals diagnosed with prediabetes to encourage routine monitoring to prevent disease progression.^12^,^16^,^47^

Quality of patient-provider communication was consistently associated with adherence across both racial groups. Men reporting higher scores on conversation quality and engagement scales were significantly more likely to adhere to blood glucose testing. The association between patient-provider communication and testing adherence is a key finding, suggesting that high-quality clinical interactions, specifically those that facilitate joint decision-making, may be an important correlate of patient engagement and adherence in this population.^48^,^49^ However, while perceived physician conversation quality and joint decision-making were associated with testing adherence in bivariate comparisons, engagement during physician visits remained in the reduced multivariable models. This pattern may indicate that active patient participation, such as preparing questions, discussing illness-related concerns, and clarifying medication purpose, is more proximally related to completing recommended testing than global perceptions of communication quality, consistent with the Chronic Care Model emphasis on informed, activated patients and productive patient-provider interactions.^26^ This pattern may also partly reflect conceptual overlap between the communication and engagement scales, with the engagement measure capturing more actionable patient behaviors related to personal agency and chronic disease self-management.^26^,^30^

Medication non-adherence was significantly associated with blood glucose testing non-adherence. Nearly half of participants reported reasons for not adhering to prescribed medications, including skipping doses, stopping when feeling better, or discontinuing due to side effects. These patterns suggest barriers beyond simple refusal, such as lack of symptom recognition, concerns about medication effects, and insufficient provider guidance.^50^ Addressing these barriers through patient education, regular follow-up, and tailored interventions may improve both medication and screening adherence.^50^

Tobacco use was strongly associated with non-adherence to annual blood glucose testing. Nearly one-third of participants reported recent tobacco usage, yet only 28.1% of this group had received a blood sugar test in the past year, compared to 71.9% of non-users. This finding is concerning because tobacco use is associated with insulin resistance,^51^ inflammation and oxidative stress,^2^ and diabetes progression.^51^,^52^ Although tobacco use is biologically relevant to diabetes risk and complications, smoking cessation is associated with lower risks of diabetes-related complications and is emphasized as an important component of diabetes care.^28^,^53^ Tobacco use may also serve as a marker of broader preventive-care disengagement, as current tobacco use patterns have been associated with healthcare utilization among non-Hispanic Black and Hispanic men aged 40 years and older with chronic conditions.^54^ These findings support integrating smoking cessation support into diabetes and prediabetes-related preventive care to help identify and support men who may be at risk for non-adherence to routine blood glucose testing.^53^,^54^ This supports the potential value of integrated interventions that combine culturally tailored diabetes education with robust smoking cessation support.^53^

Race- and ethnicity-specific analyses revealed both shared and unique factors associated with non-adherence. For non-Hispanic Black men, overweight status was associated with lower odds of non-adherence, and medication non-adherence and tobacco use were prominent factors. Among Hispanic men, progression to diabetes, age, and physician engagement were associated with non-adherence, with quality patient-provider communication standing out as a cross-cutting facilitator of adherence. These distinctions highlight the necessity of culturally tailored interventions that address population-specific challenges while promoting universally important elements such as effective communication.

Culturally responsive interventions for non-Hispanic Black and Hispanic men should move beyond generic education and address the social, cultural, and structural contexts that shape preventive care engagement, including social networks, trust, access barriers, and community norms around chronic disease self-management.^10^,^11^,^55^ DSMES programs may provide a practical framework for these efforts because they can address glucose monitoring, medication-taking, healthy eating, physical activity, problem solving, and risk-reduction behaviors in culturally responsive ways.^53^ For example, CHWs or promotoras could support DSMES-related outreach by explaining prediabetes risk and blood glucose testing recommendations, reinforcing medication adherence, and connecting men to tailored physical activity and nutrition programs.^56^,^57^ For Hispanic men, promotora-supported strategies may help address limited prediabetes awareness, financial barriers, language preferences, and perceived program relevance.^11^ For non-Hispanic Black men, peer-supported and community-based approaches may help build trust, address stigma or masculine norms around help-seeking, and strengthen self-management confidence.^55^,^58^ Partnerships with faith communities, church-affiliated barbershops, community centers, worksites, and other trusted local organizations may also help reach Black and Hispanic men in familiar settings where diabetes education, screening promotion, and lifestyle-focused programs can be normalized.^10^,^59^,^60^

## Limitations

This study has several limitations that should be considered when interpreting the findings, including its cross-sectional design, which precludes causal inferences and reliance on self-reported data which may be subject to recall and reporting biases. The online data collection method may have introduced selection bias toward individuals with internet access and greater health literacy. Future research should explore the use of mixed methods to capture both quantitative outcomes and patient-centered experiences. Our exclusive focus on non-Hispanic Black and Hispanic men with chronic conditions limits generalizability to other demographic groups. Adherence was operationalized as having any blood glucose test within the past year, which does not capture testing frequency. The progression from prediabetes to diabetes was based on self-reported healthcare professional diagnoses and not confirmed by laboratory testing, which may be subject to recall and reporting bias. Further, the survey instrument did not capture the diagnosis date related to prediabetes or diabetes, which prevented examining the temporal relationship between prediabetes history and self-reported progression to diabetes. The Nagelkerke R-square values for the reduced logistic regression models ranged from 0.111 to 0.185, indicating that the variables in the model only explain a modest amount of variance in the dependent variable. Future studies should incorporate additional predictor variables that may more robustly explain blood glucose testing adherence (eg., diagnosis temporality, cultural beliefs, mental health issues, access to diabetes education programs). Finally, our reliance on self-reported testing without verification through medical records or actual glucose values limits our ability to assess how effectively participants were managing their condition despite adherence to screening recommendations. Future longitudinal studies should follow men with prediabetes across repeated clinical encounters to determine whether changes in healthcare engagement, medication adherence, tobacco use, and social support precede changes in blood glucose testing adherence. Linking survey data with electronic health records, laboratory values, and appointment histories would allow researchers to track the timing of prediabetes diagnosis, repeat glycemic testing, referral to diabetes prevention programs, intervention participation, and progression to type 2 diabetes. Such designs could also test whether culturally tailored interventions improve testing adherence over time and whether improved adherence is associated with earlier risk management or delayed diabetes progression.

## Conclusion

This study examined important behavioral, clinical, and communicative factors associated with blood glucose testing non-adherence among at-risk minority men with prediabetes. Findings suggest that enhancing patient-provider communication quality may be an actionable strategy to promote screening adherence and potentially reduce diabetes progression. While patient-provider communication is important, our results indicate that it is one of several potential factors associated with adherence, which warrants further research. Integrated interventions addressing medication adherence and tobacco cessation, tailored to the cultural contexts of non-Hispanic Black and Hispanic men, may play a critical role in closing adherence gaps. Ultimately, multifaceted approaches that prioritize healthcare engagement, address concurrent health behaviors and acknowledge diversity within groups are essential for reducing diabetes disparities and improving outcomes among non-Hispanic Black and Hispanic men.

## Data Availability

The data accessed compiled with relevant data protection and privacy regulations. The dataset used during the current study is available from the corresponding author on reasonable request.
